# Child Abuse and Family Social Support: The Practice of Resolutions Approach

**DOI:** 10.3390/children12050580

**Published:** 2025-04-29

**Authors:** Annemariek J. W. Sepers, Marija Maric, Trudy M. Mooren

**Affiliations:** 1 ARQ National Psychotrauma Centre, Nienoord 13, 1112 XE Diemen, The Netherlands; t.mooren@centrum45.nl; 2 Department of Clinical Psychology, Utrecht University, Heidelberglaan 1, 3584 CH Utrecht, The Netherlands; 3 Research Institute of Child Development and Education, University of Amsterdam, 1018 WS Amsterdam, The Netherlands; m.maric@uva.nl

**Keywords:** child abuse, resolutions approach, family, social support

## Abstract

Background/Objectives: Child abuse is a devastating problem, and effective interventions are needed. Interventions incorporating social support have been found to be more effective in reducing parental abuse than those that do not. The resolutions approach (RA) emphasizes collaborating with the family’s social network. The present study aims to examine the role of social networks in RA. Methods: This report presents the cases of two families (children aged 8–18) who are alleged to have committed child abuse. A mixed-method study was conducted. Qualitative data based on in-depth interviews, and quantitative data obtained by repeated assessments following a single-case design were integrated. Incidents of child abuse were assessed before treatment, at the end of treatment, and at follow-up, using the Conflict Tactics Scales. An idiosyncratic measurement was administered every fortnight during the intervention. Results: In both families, members acknowledged the value of involving their social network and reported decreased incidents of child abuse. One family succeeded in involving the network, and in this family, aggressive behavior stopped soon after RA started. Results were maintained during follow-up. In the other family, aggression stopped after the baseline period, according to the parents, but not according to their youngest child. Conclusions: Although the involvement of social support is prescribed through the intervention protocol, several challenges hamper its realization. Recommendations are formulated for how to involve social network members in the context of family therapy when child safety is at risk. RA might be a valuable intervention to stop child abuse, but it needs further research.

## 1. Introduction

Child abuse is a pervasive and deeply harmful global concern. Estimates of its global prevalence vary widely, ranging from 0.3% to 36% [1,2]. Generally, child abuse is classified into four primary categories: physical abuse, sexual abuse, emotional or psychological abuse, and neglect [1,3]. A growing body of research highlights the long-term consequences of such abuse, demonstrating its profound and lasting impact on children’s physical health, psychological well-being, and cognitive development [3,4].

Previous research has shown that family violence can be transmitted across generations [5,6]. In fact, parents who experienced abuse or neglect during childhood are statistically more likely to engage in maltreatment of their own children compared to those without such adverse childhood experiences [7,8]. This pattern contributes to the perpetuation of a cycle of abuse across generations, highlighting the urgent need for effective interventions to disrupt this cycle. An important factor implicated in the intergenerational transmission of maltreatment is the availability of social support. Evidence suggests that mothers who successfully broke the cycle of abuse tended to have greater access to social support networks than those who did not [8]. Indeed, these findings underscore the protective role that social support may play in mitigating the risk of continued intergenerational violence.

Social support is defined as “the provision of assistance or comfort to others, typically to help them cope with biological, psychological, and social stressors” [9]. In the context of family interventions for child abuse, social support refers to the engagement of family members’ existing network, such as neighbors or extended family. A family’s network can provide both emotional and material support to the family alleged of child abuse [10,11]. Sociological research distinguishes between received and perceived social support. Received support refers to objective, observable interactions between people. Perceived support, on the other hand, refers to how these interactions are experienced and valued by the recipient. There may be a discrepancy between the two types. The perception of social support proves to be more important for individual well-being than the actual reception of social support. Perceived social support protects against psychopathology, while the benefits of received social support remain more contradictory [12,13].

To effectively prevent child abuse, evidence-based interventions are essential. Over the past decades, numerous interventions have been developed, with their number steadily increasing. A recent meta-analysis reported on the outcomes of 123 interventions aimed at addressing child abuse [14]. Some of these interventions are designed for prevention within the general population or among groups at elevated risk (preventive interventions), while others specifically target families in which abuse has already been identified (curative interventions).

These interventions vary in their underlying mechanisms. For instance, some focus on enhancing parenting skills, reducing parental psychological distress, or strengthening emotional and social support by activating the family’s social network. The aforementioned meta-analysis examined which of these mechanisms contributes to intervention effectiveness. Despite the availability of multiple approaches, overall effectiveness remains modest. The meta-analysis reported a small-to-moderate overall effect size (d = 0.36, *p* < 0.001), suggesting the limited impact of current interventions. Notably, interventions that incorporated social and emotional support were more successful in reducing abusive and neglectful parenting behaviors compared to those that did not. This finding highlights the importance of using emotional and social support from the social network in addressing child abuse [14].

The resolutions approach (RA) is an intervention for child abuse that uses the family’s social network to organize both emotional and social support for the parents [15]. RA has been developed by Susie Essex, John Gumbleton, and Colin Luger at the National Society for the Prevention of Cruelty to Children in Bristol [15] and is inspired by solution-focused and narrative family therapy models [16]. The aim of RA is to stop and/or prevent child abuse in families with children between 0 and 18 years old, where parents are alleged to have committed child abuse and deny the allegations. In RA, involving the network is considered a crucial element to achieve safety for the child. Within RA, it is considered a higher priority that the members of the family’s social network take joint responsibility for the child’s safety than that (one of) the parents confess to the abuse. A key aspect of joint responsibility is that network members are aware of concerns regarding the family and of the circumstances in which risk for acts of child abuse might occur. In those moments, they can offer assistance. The aim of collaborating with the network is to increase awareness about safety in the family in a wider context and to involve persons from this network in the implementation of a safety plan. Family members may indicate such persons, based on who their children trust and who is deemed suitable according to Child Protection Services [15].

### The Present Study

In the current study, our goal is to achieve a better understanding of the benefits of social support as part of RA in clinical practice with families that deal with child abuse. We describe and illustrate the application of RA in two families. Specifically, our questions are as follows: Were members of the social network identified and involved? Which role(s) did they perform? How was the RA process perceived by the family members? For the two family cases, we also investigated whether incidents of violence in the family declined during the RA intervention.

Our study is a part of a multiple single-case study (*N* = 10 families) concerned with the effectiveness of RA. The design of this study has been described elsewhere [17]. The study has been approved by the Ethical Board of the Institute of Psychology (University of Amsterdam). All families were informed about the aims of the study and the procedure, and parents and children above 12 years of age provided their active informed consent before participation. Participation was entirely voluntary, and participants did not receive any incentives or monetary compensation. They were offered RA by mental health centers specializing in the treatment of the perpetrators and victims of child abuse.

## 2. Materials and Methods

### 2.1. Participants

The families described in this study were the first two families included in the above-mentioned study in which all family members wanted to participate. For the families in this study, specific signs of child abuse were noticed by at least two informants, however, the child abuse was (partially) denied by one of the parents. Furthermore, the families and their case managers agreed to work with RA. According to Dutch law, social workers must report instances of child abuse to Child Protection Services whenever they are aware of it. A child protection officer was thus involved with the families included in this study so that the child abuse could be addressed in consultation with child protection.

### 2.2. Design

In this study, a Single Case Design was used. Family A was the first family included in the study and served as a pilot to test the research battery. After treatment and measurement of this family, we decided to add biweekly idiosyncratic measurements (IMs) and a baseline measurement (T0). As a result, the measurement moments slightly differ between the two families. For Family A, extensive measurements were taken before and after treatment (T1, T2), as well as at follow-up three and six months after treatment (T3, T4). For Family B, extensive measurements were taken 13 weeks before treatment (T0), before and after treatment (T1, T2), and at follow-up three and six months after treatment (T3, T4). Additionally, for Family B, a short IM was used to monitor the frequency of incidents of child abuse and domestic violence on a biweekly basis during the treatment phase (T1–T2).

### 2.3. Intervention

RA is an intervention to stop child abuse that explicitly uses the family’s social network to work toward a safe environment for the children [15]. RA consists of seven phases, including 20 sessions spread over a period of 12 to 18 months [15]. A form on which concerns about the safety of the child can be noted is added to this protocol [18]. During the first phase (the preparation phase), the referrer and the parents are informed about the intervention. Both parents and the referrer must agree to work on safety in the family using the social network.

In the second phase, a cooperative relationship with the parents is established, and the social network will be engaged. We follow the recommendations of the authors of RA to find network members [15]. Parents are asked to nominate network members they consider suitable to contribute to their family’s safety. The professional network checks the suitability of the persons mentioned. Members of the network are informed of the approach. The children have regular talks with their own counsellor, although this is not specifically prescribed in the RA protocol.

During the third phase, parents and the RA professional make a pictorial story in which four questions are addressed: Who’s worried? What are they worried about? What happened then? What are we going to do about it? This document describes the behavior of which the parents are accused. The child protection officer is involved in this process. The purpose of the document is to inform both the children and the network members of the concerns regarding the safety of the children in this family.

Based on the pictorial story, in the fourth phase, a step-by-step plan is made to create safety for the child in the family. Parents, their social network, and the professionals are involved. The role of the network members is specified, and safety agreements are made. Network meetings with parents, their social network, and professionals are held approximately once every six weeks to discuss whether the participants are complying with the safety agreements or whether those agreements need to be adjusted.

In the next phase, a role-playing exercise is used—the family role play. In the family role play, parents take on the role of another family in which parents acknowledge child abuse. The therapist interviews the parents about the dynamics behind the abuse and the consequences for the child. The purpose of this intervention is to increase knowledge about the behavior of the alleged perpetrator and the effects of this behavior on the child without needing to acknowledge the parents’ own involvement in such dynamics.

Finally, a definite safety plan is made, and the roles of the network members are specified in this plan. Follow-up sessions are held three and six months after the last session to evaluate the implementation of the safety plan [15].

### 2.4. Instruments

#### 2.4.1. Semi-Structured Interview

A semi-structured interview was conducted with family members at the post-treatment stage. The interview was developed based on earlier work by Gumbleton [16]. The aims were to ascertain whether network members had been identified and engaged, to understand the role of the network members in relation to child safety, and to gain insight into the perceptions of both parents and children regarding the involvement of the RA process. Questions asked for example were “(i) What did you hope to achieve by attending to RA? (ii) What did you achieve? (iii) What helped best? The participants were interviewed in face-to-face sessions. Trained research assistants not involved in the treatment conducted the interviews and transcribed the recordings. To ensure confidentiality, names were replaced by the role of the person (friend, therapist, etc.).

#### 2.4.2. Conflict Tactics Scales

For both families, the frequency of incidents of child abuse and domestic violence was assessed by the Conflict Tactics Scales (CTSs) [19]. The CTSs consist of (1) the Revised CTS (CTS2); (2) the CTS Parent–Child/Child–Parent (CTS-PC/CP); and (3) the CTS Parent–Parent (CTS-PP) [19]. CTS2 is a self-report instrument measuring psychological, physical, and sexual violence and injuries due to fights with the (ex-)partner. We use four subscales: psychological aggression, physical assault, injury, and sexual coercion. For each scale, there are two scores, one for the aggression the person used against the partner and one for the aggression used by the partner. Parents scored their behavior on an eight-point scale. The Dutch version has been found to have acceptable reliability, with alpha coefficients ranging from 0.65 to 0.82 [20].

The CTS-PC/CP is a questionnaire that assesses physical and psychological child maltreatment. Parent and children report the frequency of the occurrence of specific disciplinary behaviors. The questionnaire has 21 items divided into three subscales (physical assault, psychological aggression, and nonviolent discipline), scored on an eight-point scale. The reliability indices demonstrate a range from low to moderate reliability. Additionally, evidence was found for construct and discriminant validity of the subscales, with alpha coefficients ranging from α = 0.55 (Physical Assault) to α = 0.60 (Psychological Aggression) [19]. The Physical Assault scale demonstrates low internal consistency, due to the inclusion of diverse forms of severe violence—ranging from more common behaviors (e.g., hitting) to rare incidents (e.g., burning). As individual parents are unlikely to engage in all types, item correlations within the scale are reduced [19].

The CTS-PP is a questionnaire derived from the CTS2 in which children score the frequency of aggressive behavior between their parents. The questionnaire has 19 items, scored on an 8-point scale. To our knowledge, no reliability indices are known. Full details of the measures used can be found in the study protocol [17].

#### 2.4.3. Idiosyncratic Measurement

In addition to the CTS scales, an Idiosyncratic Measurement (IM) was used for Family B. The IM is a personalized questionnaire consisting of 5–10 items derived from the CTS questionnaires, filled in during T0 and on a biweekly basis. The IM represents the frequency of aggressive acts in the family. Parents and children were asked to complete the IM.

#### 2.4.4. Subjective Units of Safety Scale

The subjective perception of safety was assessed using the Subjective Units of Safety (SUS) scale. A SUD score is a self-reported and subjective measure used to assess distress on a ten-point scale [21]. During network meetings, all members of the families individually rated safety within the family on a scale ranging from 0 (recurrence of similar or worse abuse/neglect is certain) to 10 (no unsafety in the family at all). These meetings took place approximately once every six weeks.

### 2.5. Analysis Plan

#### 2.5.1. Social Network

The semi-structured interviews were analyzed using MAXQDA 2022 [22]. We based the analyses on a directed content approach, which is a way of analyzing qualitative data using both inductive and deductive methods [23,24]. The interviews were read in full by the first author. She coded the text parts that were about the professional and personal network. In these sections, predetermined axes were used (perception, role, experience of RA). Further codes were developed within these axes through open coding. After coding all the interviews, the coding of the first two interviews was repeated to ensure the codes found in the last interviews were also applicable to the first interviews. This process was repeated by the co-author (TM). Differences were discussed until a consensus was reached. Finally, the findings were interpreted within the research team.

#### 2.5.2. Incidents of Child Abuse

To investigate whether incidents of child abuse and domestic violence in these families declined during the RA intervention, we used the data from the CTS and the SUS scales. For Family B, we could additionally use the IM data. These were presented graphically, a procedure frequently used when analyzing SCD data [25], and furthermore, the IM data were analyzed using the nonoverlap of all pairs (NAP) index. The NAP index represents the probability that the score from the first half of the treatment is higher than the score from the second half of the treatment [26]. The effect can be interpreted as weak (NAP index 0.50–0.65), medium (NAP index 0.66–0.92), or large (NAP index 0.93–1.0). An effect lower than 0.50 may indicate a reverse effect—that is, the aggression increases.

## 3. Results

### 3.1. Case Illustration

#### 3.1.1. Family A

Family A consists of a single mother (age 49) and her 11-year-old child; the father is unknown. The child and another family member accused the mother of using physical violence against the child. The mother denied all allegations. The school reported irregular attendance by the child. Because of the severity of the allegations, the child was placed with a foster family. The mother was reluctant to co-operate with the child protection agency because she felt wrongly accused. In order to regain custody, she agreed to take part in RA. Child protection services also agreed to give her a chance to regain custody. A court-appointed child protection guardian, a family therapist, the mother’s psychotherapist, the foster care counsellor, and the RA therapist were all involved in the RA process.

*Intervention*. In the preparation phase, we discussed with the mother the need to involve the social network. She was very willing to discuss this topic, although she did not have an active network. It appeared that some family members were accused of abusing children themselves: these members were not invited to participate in the RA process. At the start of the intervention, the mother had no active contact with her neighbors; however, she was very willing to invite them to take part in the process. In the second phase, the engagement phase, a cooperative relationship was established with the mother and the social network was engaged. A neighbor, two former friends, and a niece were willing to assist the family in the RA process. The mother shared that in her youth, she was severely abused by her parents. She admitted that she sometimes “taps” her child in order to discipline him. However, she objected to the notion that she was maltreating her child.

During the third phase, mother and the RA professional created the words and pictures document and presented this to the child and the social network members. In the fourth phase, the preliminary safety plan for Family A was made. The safety plan contained several steps. The first step was that mother and child met weekly for a few hours under the supervision of the guardian. During these meetings, the guardian got an impression of how the mother responded to the child’s disobedience. The guardian observed a positive interaction between mother and child. For instance, the mother interacted according to his needs. At the same time, she seemed less able to discipline him in a positive way. A youth service worker worked with the mother in order to break this negative cycle. During the next step, mother and child met under the supervision of a member of the social network. This step also went well, and the mother made progress in disciplining her child. Subsequently, the child was increasingly at home with the mother.

Finally, a definite safety plan was made, and the child left the foster family in order to live at home with mother again. Prior to the intervention, the mother had no contact with individuals within the network or in the neighborhood. During the RA process, the network became engaged in several different ways, as described in the safety plan; the two friends and niece provided childcare for one afternoon and evening per week, allowing the mother to pursue her own activities. In the case of a challenging situation, the mother could contact her friends, niece, or neighbor by phone, and the neighbor could come to assist the mother. In case of feeling unsafe, the child had the option of contacting the neighbor or seeking assistance from her directly. The neighbor would come to discuss with the mother what could be done. In this family, six of the seven steps of RA were followed. The use of family role play proved to be unnecessary as, during the engaging phase, the mother acknowledged the abuse.

#### 3.1.2. Family B

Family B consisted of two children (aged 16 and 13), their father (aged 47), and their mother (aged 44). The father was diagnosed with post-traumatic stress disorder (PTSD) following a military peacekeeping mission. He had undergone several trauma-focused treatments, so far with unsatisfying results. The father used violence in the home (throwing objects) and emotionally abused the children (screaming and yelling at them, calling them names). He did not deny the aggression but could not acknowledge the harmful effects of his behavior on the children. In his view, such an upbringing was necessary for children to survive in today’s society. Although the mother disagreed, she was unable to stop the father’s aggression; she observed that both children often retired to their rooms. The mother stated clearly that she wanted a safer environment for the children and threatened that she and the children would leave the father if his behavior did not change. In order to stay together as a family, the parents agreed to take part in RA. In the past, the father had undergone several trauma-focused treatments, but so far, these had not reduced the violence in the family. Moreover, family members had not been involved. Although the family has relatives and neighbors, they tend not to socialize with them. A child protection guardian, the father’s psychotherapist, and the RA therapist were involved in the RA process. In agreement with professionals and parents, the following goals were set: the home environment will be a safe environment for the children without the father throwing objects, screaming, yelling, or calling names.

*Intervention*. In the preparation phase, RA was explained to the family members and the professional network. The professional network of this family consisted of the father’s therapist and the child protection officer. Furthermore, the importance of involving the social network as part of RA was conveyed to both parents. In the engagement phase, a cooperative relationship was established with the parents and the social network was further explored. We discussed with them that an important aspect of RA is to involve the social network. The social network may have a role in safeguarding the children. The parents explained that the family lived in a very isolated way. Although they had a social network (parents, brothers and sisters, neighbors, colleagues, parents of the children’s friends), they had no contact with them because the father had become very suspicious of people since he attended the peacekeeping missions. We discussed this topic with the child protection officer. Surprisingly, the child protection officer agreed with the parents that it was not necessary to involve social network members. As the social network could not be engaged, the safety plan remained limited. The safety plan for this family consisted of the agreement that the father would leave the family at times when he felt more aroused and irritable; such moments were at times predictable, for example, on commemoration days related to the war in which he had fought. On these days, he hired a holiday cottage. On other, less predictable moments, he would go for a walk. The words and pictures document was made and presented to the children. The use of family role play proved to be unnecessary as, during the engaging phase, the father acknowledged the influence of his aggressive behavior on the children.

### 3.2. Involvement of the Social Network

#### 3.2.1. Family A

Although the mother had no contact with her social network prior to the intervention, the mother and the RA therapist successfully involved four network members—a neighbor, two former friends, and a niece—in the RA process and provided them with an ongoing role in this family. The network members were informed about the concerns regarding the family by the mother and the RA therapists using the word and pictures document.

In the interview, the mother described how these network members played an important role for her as well as for her child. The mother experienced much support from her network members: “They support me, they back me up. They know the whole story. I can talk to them about it. They ask about it. They support me emotionally.” The child was able to contact the network members whenever he did not feel safe. The mother reported that there was always a phone available so he could call whenever he needed. The mother said that the child used this opportunity at the beginning of the RA process but had done so less often recently.

#### 3.2.2. Family B

Although the social network could not be involved with this family, the mother saw benefits in involving the network. According to her, advantages of the involvement of the network members could have been that “You are better looked after” and “Others know my story”. Consequently, the role of the network members could have been to provide emotional support to the parents and children: the children could have had someone outside the family with whom they could talk about the aggression in the family. This family included a time-out procedure for the father in their safety plan. Network members could have provided a time-out place in the form of a room in their house, their holiday house, or a camper.

### 3.3. Perception of the RA Process

#### 3.3.1. Family A

The mother said she disagreed with the accusation of child abuse. She felt initially that no one listened to her, and she felt she was being antagonized. This changed over the course of the RA process. Gradually, she experienced that the professionals involved in RA, and the family guardian, were committed to her, listened to her, trusted her, and advocated for her in court. Moreover, she could and did call them when she had problems with her child’s behavior and was given advice on how to address the problem. The child liked that he had his own counsellor with whom he spoke regularly.

#### 3.3.2. Family B

The mother experienced and appreciated more control from agencies outside the family and therefore more protection: “All these years you are in such a situation, and you do get a bit isolated by it. Then suddenly people know, and you are no longer alone […] There has been more control, not control in the sense of pointing a finger […] I feel more protected, that someone is backing me.” Moreover, she noticed that there was more pressure on her partner to change his behavior. He became more aware of his behavior and its consequences for the children, and he subsequently used less aggression: “He is no longer so quick to lash out, and he thinks better before he does or says something.” She also saw a change in the elder child: he participated more in family activities and retreated less to his own room. The younger child also found it helpful that he was now more involved in the counselling process. He especially liked having his own counsellor with whom he could talk, and he felt that RA was helpful because, since starting RA, he cried less. The mother felt that the safety plan was working. She explained that she used to be afraid of her partner, but that because of the safety plan, she had gotten a better grip on the situation. Indeed, the father withdrew at the agreed cue. He liked that he could easily get in touch with the RA workers. If he or other family members called, their calls were returned quickly. He also noticed that his children liked having their own counsellor. The father felt that RA helped his family but did not help him with his PTSD symptoms.

### 3.4. Incidents of Child Abuse

#### 3.4.1. Family A

Conflict tactics scales. For Family A, the CTSs were administered before and after treatment (T1, T2), and at follow-up at three and six months after treatment (T3, T4). The mother reported on the CTSs (Table 1) that she used psychological aggression against her partner in the year prior to the intervention. On the post-treatment measurements and during the follow-ups (T2, T3, and T4), she indicated that there was no longer any psychological aggression. The mother indicated that she did not use any form of violence towards her child; however, the child reported on the CTSs (Table 1) that the mother had used psychological aggression frequently (11–20 times) and physical aggression infrequently (3 times) against him in the year prior to the intervention. He indicated that after treatment (T2) and at follow-up (T3), all forms of aggression had stopped. At follow-up, the CTS outcomes revealed that all forms of aggression had stopped, as reported by both mother and child (Table 1).

*SUS scale during network meetings*. Safety was rated during the network meetings. The scores showed that, according to all members, safety had increased during the intervention (Table 2).

#### 3.4.2. Family B

Conflict tactics scales. The CTS (Table 3) showed that, according to the family members, in the year prior to the intervention (T0), psychological and, to a much lesser extent, physical aggression occurred between the parents. The father indicated that he did use psychological aggression against both children, and the children also mentioned that psychological aggression was used against them. Only the younger child signaled infrequent physical aggression directed at him.

According to all the family members, the physical aggression between the parents stopped right at the start of the intervention (T1). However, the father mentioned that he still used psychological aggression against both children at the start of treatment. After treatment and during the follow-up measurements, this no longer occurred, he noted. According to the elder child, all aggression had stopped at the end of the treatment. The younger child, however, still signaled psychological aggression at the end of the treatment.

Follow-up measurements were conducted five and nine months after treatment. The parents and the elder child no longer reported any aggression at both follow-up moments. However, according to the younger child, the physical aggression stopped at follow-up, although he noticed some psychological aggression at the first follow-up measurement, which had stopped at the second. The violence between the parents, according to the younger child, decreased but was still present during the last follow-up (Table 3).

SUS scale during network meetings. All the professionals rated that safety had increased over the course of treatment (Table 4). The parents, on the other hand, initially gave a high score for safety in the family, but these scores dropped after the start. At the end of the intervention, safety had increased.

**Figure 1:**
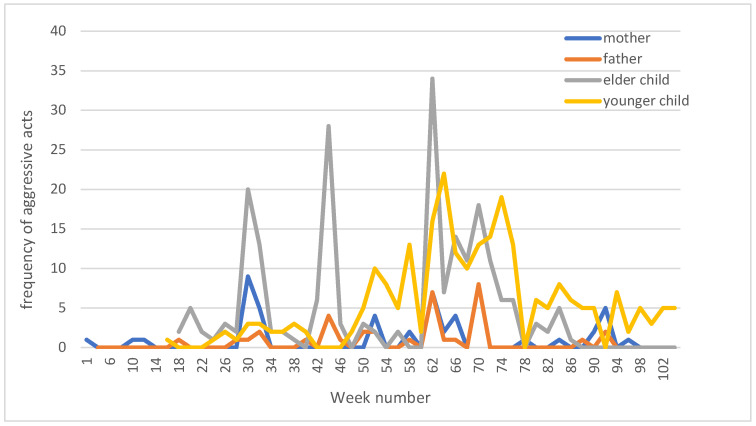
Bi-weekly frequency of aggressive acts in Family B.

Idiosyncratic measurement. For Family B, a short IM was used to monitor the frequency of incidents of child abuse and domestic violence on a more frequent (biweekly) basis during the treatment phase (T1–T2). The IM is a personalized questionnaire consisting of five to ten items derived from the CTS questionnaires filled in by parents and children during T0. The IM represents the frequency of aggressive acts in the family. The NAP index calculated for the IM (Figure 1) showed a different picture from the CTS measurements. Only the father’s scores revealed a decrease in aggressive behavior in the second half of treatment compared to the first half (NAP index 0.57, indicating a weak effect). The scores of the mother and elder child showed no effect (NAP index 0.40, 0.48), and the scores of the younger child showed an increase in aggression in the second half of the treatment (NAP index 0.19).

## 4. Discussion

Child abuse is a severe and devastating problem worldwide, and it can have serious long-term consequences for children’s development and well-being [3,4]. Child abuse can be transmitted across generations, perpetuating cycles of maltreatment within families [5,6]. A lack of social support might increase the risk of child abuse and neglect [27]. Conversely, the presence of strong social support networks may play a critical role in disrupting this intergenerational cycle [6]. Making use of the social networks surrounding families could therefore increase the effectiveness of interventions that aim to stop child abuse [28,29].

In this study, we investigated the involvement of a social network as part of RA. We explored its significance in the process of enhancing safety in two case examples. We described the treatment procedure and how it was evaluated, and we studied the role network members performed. Furthermore, we analyzed whether incidents of child abuse and domestic violence declined during RA in the two families.

Our findings show that, despite Family A initially having a limited social network and a history of child maltreatment within the family, suitable network members were successfully identified—aligning with the findings of Armstrong et al. [30]. In contrast, attempts to engage the social network in Family B were unsuccessful, despite the family’s broader network. In this regard, it is notable that the child protection officer complied with the parents’ decision not to engage the network.

Several underlying processes may have contributed to the failure to mobilize social support in this case. Firstly, for the parents, shame and reluctance to admit violence toward family members may have contributed. In families suspected of child abuse, there may be resistance to involving the network because people do not want to reveal openly that domestic violence is occurring in the family [31]. Secondly, the mother had revealed that she was afraid of her partner. Like the mother, social workers may also feel threatened or anxious when interacting with people who use violence. As a result, they may go along with the parents’ desire not to involve the network, and thus avoid the confrontation. Whenever parents do not want to involve the network, do RA professionals have sufficient authority to bring this into effect? All this stresses the importance of different organizations collaborating around families. Turnell and Essex [15] also emphasize the importance of supporting the secure parent. By not involving the network, the professionals may have succumbed to the parent who used aggression instead of supporting the non-aggressive parent. In this way, the protocol was not fully implemented, and the intervention might not have worked as well as it could.

In both families, family members and professionals perceived positive effects of RA on family safety. The family members felt more supported and more protected; they stated that the safety plan increased family safety. In Family A, the quantitative results demonstrate a clear picture; mother and child agree that there is no longer aggression after treatment. In contrast, for Family B, the quantitative measurements revealed some discrepancies. Firstly, the results showed more aggression on the biweekly than on the monthly measurements. A possible explanation for the difference between the biweekly and monthly measurements is that it is easier to remember events for a shorter period than for a longer period. Reporting over a shorter time might thus give more precise observations than reporting over a longer period. Secondly, in Family B was a difference in how parents scored the occurrence of aggression and how the child did. Although the mother reported positive results, the question remained as to whether (verbal) aggression had completely stopped in this family.

Furthermore, in both families, parents initially gave high scores for safety during the network meetings, whereas low scores were expected at the start of treatment. This could be due to two factors. Firstly, at the beginning, parents may not have had much confidence in the new intervention and wanted to prove that it was safe for the children in their family, thus giving high scores for safety. Secondly, during the process, parents became more perceptive about what is seen as aggression by professionals.

Our first recommendation is that it is important for the professional team to be united on the necessity of involving the network and not to loosen this principle. However, engaging the network can be a challenge, so professionals may need more guidance. Although the NICE guideline for Child Abuse and Neglect [27] states that a lack of social support may make a family more vulnerable to child abuse and neglect, the guideline does not prescribe how professionals can build and engage the network or what role the network members could have. We recommend that this be addressed in future revisions of the guidelines.

Secondly, the RA protocol is not particularly clear about whether the children should also be spoken to regularly without their parents. During the treatment of these families, there were supplementary individual sessions for the children. The children interviewed mentioned that they really appreciated these sessions. Furthermore, because network members can provide support in an emotional and practical sense to the children, and children are important informants about the safety in the family, particular care should be taken to ensure that children are heard and involved in the RA process. It is important that they are also aware of the safety agreements, which can be communicated to the child through words and pictures, in the same way as concerns earlier in the process were discussed with the children.

### Limitations

A strength of our study is that it gathers information from a diverse range of individuals, including the children who experienced the abuse. A limitation of this study is that the network members themselves were not explicitly interviewed about their role and involvement. This could have provided considerable additional information about how they interpreted their role and how the individual members fulfilled it. Another limitation is the small sample size, as the study focuses on only two families. Additionally, the timing of data collection was not consistent across both cases, which influenced the comparability of the findings.

## 5. Conclusions

In conclusion, this study underscores the importance of involving the social network in RA. It illustrates that in both families, members acknowledged the value of involving their social network and reported decreased incidents of child abuse. One family succeeded in involving the network, and in this family, aggressive behavior stopped soon after RA started. Results were maintained during follow-up. In the other family, aggression was said to have stopped after the baseline period according to the parents, but not according to their youngest child. While engaging the network is experienced as being helpful, collaborative engagement is needed to realize it. It is clear that there are challenging dynamics in offering services to families dealing with child abuse. RA might be a valuable intervention to stop child abuse, but it needs further research.

## Figures and Tables

**Table 1:** Type and frequency of incidents of child abuse and domestic violence at pre-treatment, post-treatment, and follow-ups for Family A.

	T1 ^1^	T2 ^2^	T3 ^3^	T4 ^4^
**Mother**				
**CTS2 Self**				
Psychological Aggression	14	0	0	0
Physical Assault	0	0	0	0
Injury	0	0	0	0
Sexual Coercion	0	0	0	0
**CTS2 Partner**				
Psychological Aggression	^a^	0	0	0
Physical Assault	1	0	0	0
Injury	0	0	0	0
Sexual Coercion	1	0	0	0
**Mother about child**				
Frequency of Psychological Aggression (CTS-PC)	^a^	1	0	0
Frequency of Physical Assault (CTS-PC)	^a^	0	0	0
**Self-report child**				
Frequency of Psychological Aggression (CTS-CP)	17	0	0	^a^
Frequency of Physical Assault (CTS-CP)	3	0	0	^a^
Frequency of aggressive behavior between parents (CTS-PP)	4	^a^	^a^	^a^

^a^ missing; ^1^ T1, start intervention, aggression in a year; ^2^ T2, after intervention, aggression in a month; ^3^ T3, 5 months after intervention, aggression in a month; ^4^ T4, 9 months after intervention, aggression in a month.

**Table 2:** Subjective unit of Safety Scores ^1^ during network meetings—Family A.

Session Number	1	2	3	4	5	6	7	8	9	10	11
Mother	5	4	5	6	6	6	7	7	7	NA	7
Guardian	2	3	3	4	5	5	6	5	6	NA	7
Mother’s therapist	^a^	^a^	^a^	6	6	6	7	7	8	NA	8
Family therapist	^a^	^a^	^a^	5	5	6	7	6	7	NA	7.5
Foster care officer	4	4	4	NA	5	6	6	6	7	NA	^a^
RA therapist	2	3	5	5	5	6	7	6	7	NA	7.5
Mother’s friend 1	^b^	^b^	^b^	^b^	^b^	^b^	^b^	^b^	^b^	NA	^b^
Mother’s friend 2	^b^	^b^	^b^	^b^	^b^	^b^	^b^	^b^	^b^	NA	^b^

NA; not available, assessment missing; ^a^ not participating in the RA process; ^b^ Network member invited but absent; ^1^ SUS range from 0 (Recurrence of similar or worse abuse/neglect is certain) to 10 (No unsafety in the family at all).

**Table 3:** Type and frequency of incidents of child abuse and domestic violence at baseline, pre-treatment, post-treatment, and follow-ups for Family B.

	T0 ^1^	T1 ^2^	T2 ^3^	T3 ^4^	T4 ^5^
**Mother**					
CTS2 Self					
Psychological Aggression	4	0	0	0	0
Physical Assault	0	0	0	0	0
Injury	0	0	0	0	0
Sexual Coercion	0	0	0	0	0
CTS2 Partner					
Psychological Aggression	16	0	1	0	0
Physical Assault	0	0	0	0	0
Injury	0	0	0	0	0
Sexual Coercion	0	0	0	0	0
**Mother about eldest child**					
Frequency of Psychological Aggression (CTS-PC)	1	0	0	0	0
Frequency of Physical Assault (CTS-PC)	0	0	0	0	0
**Mother about youngest child**					
Frequency of Psychological Aggression (CTS-PC)	0	0	0	0	0
Frequency of Physical Assault (CTS-PC)	0	0	0	0	0
**Father**					
CTS2 Self					
Psychological Aggression	22	0	^a^	0	0
Physical Assault	2	0	^a^	0	0
Injury	0	0	^a^	0	0
Sexual Coercion	0	0	^a^	0	0
CTS2 partner					
Psychological Aggression	21	0	^a^	0	0
Physical Assault	0	0	^a^	0	0
Injury	0	0	^a^	0	0
Sexual Coercion	0	0	^a^	0	0
**Father about eldest child**					
Frequency of Psychological Aggression (CTS-PC)	25	10	^a^	0	0
Frequency of Physical Assault (CTS-PC)	0	0	^a^	0	0
**Father about youngest child**					
Frequency of Psychological Aggression (CTS-PC)	15	1	^a^	0	0
Frequency of Physical Assault (CTS-PC)	0	0	^a^	0	0
**Self-report eldest child**					
CTS-PC					
Frequency of Psychological Aggression (CTS-CP)	9	10	0	^a^	^a^
Frequency of Physical Assault (CTS-CP)	0	0	0	^a^	^a^
Frequency of aggressive behavior between parents (CTS-PP)	6	1	0	^a^	^a^
**Self-report youngest child**					
CTS-PC					
Frequency of Psychological Aggression (CTS-CP)	4	0	7	1	0
Frequency of Physical Assault (CTS-CP)	2	0	0	0	0
Frequency of aggressive behavior between parents (CTS-PP)	20	0	12	5	1

^a^ missing; ^1^ T0, start baseline period, aggression in a year; ^2^ T1, start intervention, aggression in a month; ^3^ T2, after intervention, aggression in a month; ^4^ T3, 5 months after intervention, aggression in a month; ^5^ T4, 9 months after intervention, aggression in a month.

**Table 4:** Subjective unit of Safety Scores ^1^ during network meetings—Family B.

Session Number	1	2	3	4	5	6	7	8	9	10	11	12	13	14
														
Mother	6	NA	6	4	4	6	5	NA	NA	8	8	7	NA	8
Father	7	NA	7	5	4	5	^a^	NA	NA	7	^a^	7	NA	7.5
Eldest child	^b^	^b^	^b^	^b^	^b^	^b^	6	^b^	^b^	^b^	^b^	^b^	^b^	^b^
Youngest child	^b^	^b^	^b^	^b^	^b^	^b^	6	^b^	^b^	^b^	^b^	^b^	^b^	^b^
Father’s therapist	4	NA	4	4	4	5	4	NA	NA	5	7	7	NA	NA
RA therapist	4	NA	4	NA	4	5	4	NA	NA	5	6	6	NA	NA
Doctor child protection	^b^	^b^	^b^	^b^	^b^	^b^	^b^	^b^	3	^b^	6	^b^	^b^	^b^

NA; not available, assessment missing; ^a^ absent; ^b^ Network member invited but absent; ^1^ SUS range from 0 (Recurrence of similar or worse abuse/neglect is certain) to 10 (No unsafety in the family at all).

## Data Availability

During the research, all data will be stored on the academic treatment center storage that complies with the academic center’s ICT Security Policy and provides for regular backups and access control.

## Abbreviations

The following abbreviations are used in this manuscript:

CTS2	Conflict Tactics Scale
CTS-PC/CP	Conflict Tactics Scale Parent–Child/Child–Parent
CTS-PP	Conflict Tactics Scale Parent–Parent
CTSs	Conflict Tactics Scales
IM	Idiosyncratic measurements
NAP	Nonoverlap of all pairs
PTSD	Post-traumatic stress disorder
RA	Resolutions Approach
SCR	Single Case Report
SUS	Subjective Units of Safety
